# The eastern migratory caribou: the role of genetic introgression in ecotype evolution

**DOI:** 10.1098/rsos.150469

**Published:** 2016-02-03

**Authors:** Cornelya F. C. Klütsch, Micheline Manseau, Vicki Trim, Jean Polfus, Paul J. Wilson

**Affiliations:** 1 Biology Department, Trent University, Peterborough, Ontario, Canada K9J 7B8; 2 Protected Areas Establishment and Conservation Directorate, Parks Canada, Gatineau, Quebec, Canada J8X 0B3; 3 Natural Resources Institute, University of Manitoba, Winnipeg, Manitoba, Canada R3T 2N2; 4 Manitoba Conservation and Water Stewardship, PO Box 28, 59 Elizabeth Drive, Thompson, Manitoba, Canada R8N 1X4

**Keywords:** approximate Bayesian computation, introgression, ecotype, secondary contact zone, conservation, species at risk

## Abstract

Understanding the evolutionary history of contemporary animal groups is essential for conservation and management of endangered species like caribou (*Rangifer tarandus*). In central Canada, the ranges of two caribou subspecies (barren-ground/woodland caribou) and two woodland caribou ecotypes (boreal/eastern migratory) overlap. Our objectives were to reconstruct the evolutionary history of the eastern migratory ecotype and to assess the potential role of introgression in ecotype evolution. STRUCTURE analyses identified five higher order groups (i.e. three boreal caribou populations, eastern migratory ecotype and barren-ground). The evolutionary history of the eastern migratory ecotype was best explained by an early genetic introgression from barren-ground into a woodland caribou lineage during the Late Pleistocene and subsequent divergence of the eastern migratory ecotype during the Holocene. These results are consistent with the retreat of the Laurentide ice sheet and the colonization of the Hudson Bay coastal areas subsequent to the establishment of forest tundra vegetation approximately 7000 years ago. This historical reconstruction of the eastern migratory ecotype further supports its current classification as a conservation unit, specifically a Designatable Unit, under Canada’s Species at Risk Act. These findings have implications for other sub-specific contact zones for caribou and other North American species in conservation unit delineation.

## 1. Introduction

Contemporary spatial genetic variation in natural populations is the result of complex relationships between evolutionary change, phylogeographical structure and gene flow [1] at different points in time. One major driving force for contemporary phylogeographical structure has been linked to repeated Pleistocene glacial cycles that led to contraction of populations and retreat into isolated refugia [2,3]. Therefore, isolation and local adaptation in refugia possibly led to the formation of different spatially distributed populations [2,3]. However, in interglacial periods, refugial populations expanded and potentially merged in secondary contact zones, with the potential for exchanging genetic material. It is increasingly recognized that acquisition of genetic material through introgression (i.e. the mixture of different lineages and gene pools where traits found in contributing parental taxonomic entities can be transferred to ‘hybrid’ offspring) may lead to selective advantages depending on environmental factors [4–6]. Studies on hybridization among species are relatively common (for reviews see [4–6]), while the divergence and subsequent secondary contact of subspecies and differentiated populations is less well studied but increasingly recognized as an important component of the evolutionary history of contemporary populations [7,8].

The need to characterize intra-specific isolation and potential merging events is especially important for wide-ranging species like caribou (*Rangifer tarandus*) because of the morphological, ecological and behavioural variation recognized in the species [9–18]. Four extant subspecies (*Rangifer tarandus groenlandicus*—barren-ground, *R. t. caribou*—woodland caribou, *R. t. pearyi*—Peary’s caribou, *R. t. granti*—Grant’s caribou) have been described in North America [9]. Importantly, the phylogenetic origin of these subspecies differs. Specifically, all but the woodland caribou subspecies originated in northern Beringian glacial refugia and expanded geographically after the last glacial maximum [3,11]. The two major lineages (i.e. woodland and Beringian, *sensu* [3]; which correspond to the North American and Beringian lineages in [12,14]) are generally accepted to have evolved in allopatry in geographically separated Pleistocene refugia north and south of the ice sheet. Further, the phylogeography of woodland caribou supports origins from three southern sublineages [3] having evolved separately from each other starting at the end of the Pleistocene.

While caribou subspecies delineation provides a foundation for establishing conservation units, specifically Designatable Units (DUs) under the Committee on the Status of Endangered Wildlife in Canada, the delineation of DUs within subspecies, that can include ecotypes [15], is more challenging. Recent genetic surveys of caribou ecotypes show extensive contemporary gene flow between migratory and sedentary woodland caribou in Quebec [15–17]. In contrast, more prominent past sub-specific interbreeding events following the last glacial maximum are found in western Canada where genetic structure does not necessarily correlate with taxonomic designations [12,13,18]. There is increasing recognition that the delineation of conservation units should consider complex population histories coupling relatively recent divergence and secondary contact with introgression in characterizing the evolutionary legacy of such units [8].

The eastern migratory caribou ecotype (DU4), found in northern Manitoba, Ontario and Quebec [15,17], contains at least three populations (George River, Leaf River and Pen Island) with a potential fourth population (Cape Churchill). Previous work [9,15,19,20] suggested that the Pen Island population may have originated from interbreeding event(s) of the two subspecies (barren-ground and woodland). Pen Island caribou are morphologically (e.g. body size, skull measurements and antler position) similar to woodland caribou, with the exception of antler characteristics and migratory behaviour that are more similar to barren-ground caribou [19,20].

Our research objective was to examine the contribution of phylogeography and potential genetic introgression in the contemporary genetic structure of the eastern migratory ecotype. Alternative to the evolutionary origin by introgression as described above, the eastern migratory ecotype may have split from either woodland caribou or barren-ground caribou with little to no admixture between subspecies, or admixture may be a relatively recent phenomenon resulting in adapted sub-specific hybrids. To test these competing hypotheses, we first characterized patterns of genetic diversity using a comprehensive survey of 10 microsatellite loci for more than 1300 caribou and 1200 mitochondrial DNA (mtDNA) control region sequences from the two subspecies and two ecotypes of woodland caribou found in Manitoba and Ontario to determine their level of distinctiveness at a phylogeographical scale, thereby focusing on the identification of higher order groups (e.g. ecotypes and subspecies). We used an approximate Bayesian computation (ABC) framework [21] to identify the most supported evolutionary model explaining the history of the eastern migratory ecotype. With this, we determined the time frame of divergence and any contemporary or historic admixture events that contributed to the evolutionary history of this ecotype. Finally, we compare our findings to other published caribou genetic studies across Canada and discuss the ABC results in light of the Late Pleistocene ice sheet retreat as well as Holocene landscape vegetation changes in central Canada.

## 2. Material and methods

### 2.1 Study area and collection of samples

**Figure 1.**
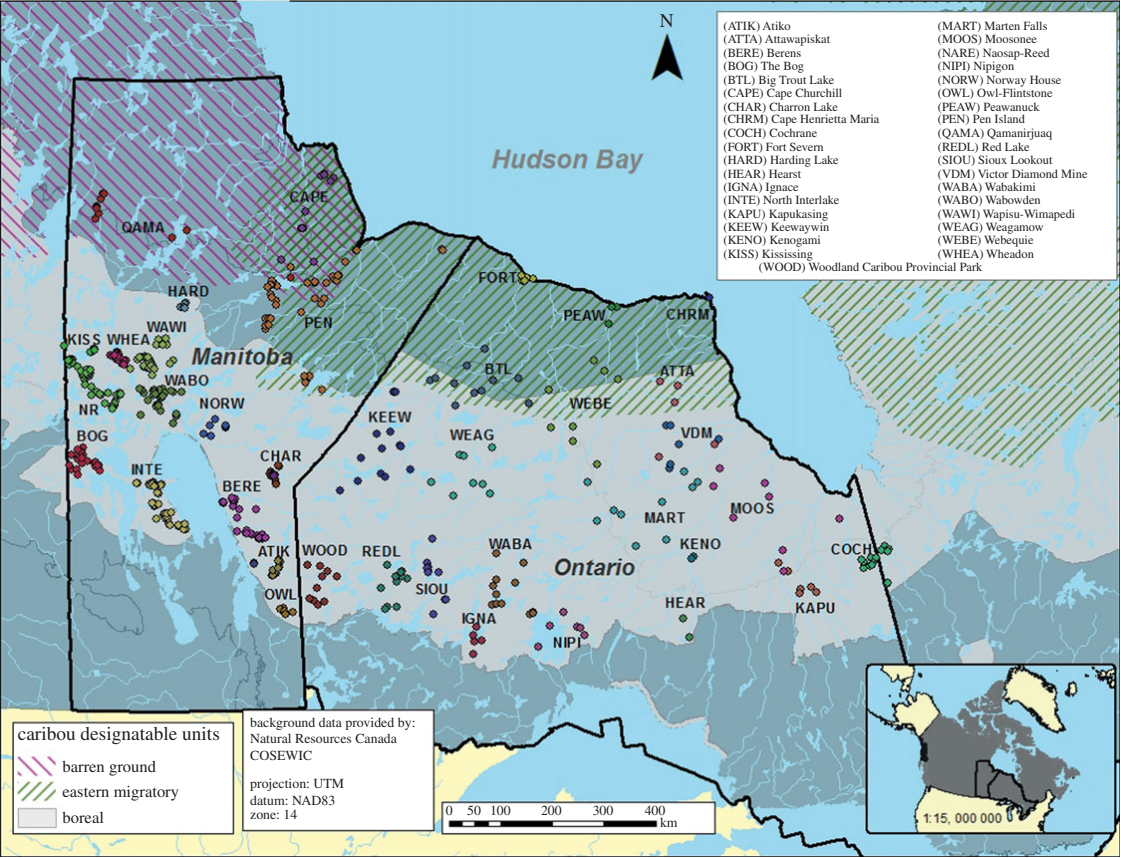
Caribou sampling locations in Manitoba and Ontario, Canada.

The study area encompassed Manitoba and Ontario, Canada. Samples were collected from barren-ground caribou (DU3 [15]; figure 1; electronic supplementary material, S1) and the two currently recognized woodland caribou ecotypes; eastern migratory caribou (DU4; Pen Island herd) in the northeastern part of Manitoba and boreal caribou (DU6) in central and southern parts of the provinces where samples were collected systematically across the entire range (figure 1; see [22–25] for more details on the sampling protocol). To ensure that fecal pellets were from the targeted groups, the collection was assisted by behavioural information (migration events and movement rates obtained from telemetry data) and group size and composition. The study area consisted of different ecozones (Taiga Shield, Hudson Plains, Boreal Shield and Boreal Plains ([20,26] and references therein); figure 1).

### 2.2 Laboratory work

#### 2.2.1 DNA extraction

DNA was extracted from fecal pellets using the protocol outlined in [22,23]. Briefly, a sterile cotton-tipped applicator (Puritan) was used to swab fecal pellets targeting the outer mucous layer in order to obtain epithelial cells for DNA extraction. Subsequently, swabs were placed into 300 μl of 1×lysis buffer. Then, a two-step digestion was carried out using 20 units of proteinase K (Roche Applied Science) in a first incubation step of 2 h at 65°C to be followed by a second digestion step using an additional 20 units of proteinase K and an incubation period of 12 h at 37°C. Next, DNA extraction was carried out using a DNeasy Blood and Tissue Kit (QIAGEN) following the manufacturer’s protocol. Finally, samples were eluted in preheated (approx. 70°C) 65.0 μl of 0.1 M TE buffer and stored at −20°C until further processing.

#### 2.2.2 Microsatellite amplification and genotyping

Extracted DNA samples were amplified at 10 microsatellite loci. Of these, six loci (Rt5, Rt6, Rt7, Rt9, Rt24 and Rt30) were taken from [27] with the reverse 5′ taken from [28] for Rt5, two markers were taken from [29] (BM848 and BM888) and one marker each from [30] (BMS1788) and [29,31] (Map2C). Amplification of all loci was conducted in three multiplex reactions containing the following primer combinations: multiplex 1: Rt6, Rt9 and Map2C; multiplex 2: Rt5 and Rt30; multiplex 3: BMS1788 and Rt7; and four singleplexes for BM888, BM848 and Rt24 [22,23]. Reactions were performed in a 7 μl volume containing: 1×PCR buffer; 2.0 mM MgCl; 0.2 μg ml^−1^ of bovine serum albumin; 0.4 μM of each primer pair (forward and reverse) according to the above-mentioned multiplex combinations; 0.2 μM of each dinucleotide triphosphate; 0.5 unit of *Taq* polymerase (Invitrogen Life Technologies) and 5 ng of DNA template. The thermocycling protocol included the following steps: a denaturation step at 95°C for 10 min, followed by 30 cycles of 94°C for 30 s, an annealing step for 60 s at 56°C for multiplex 1 (58°C for multiplex 3 and 60°C for multiplex 2 and singleplexes) and 72°C for 1 min. A final extension time of 65°C for 15 min completed the reaction. For the sexing reaction, the thermocycling protocol included a denaturation step at 94°C for 5 min, followed by 30 cycles of 94°C for 30 s, an annealing step for 30 s at 56°C and an extension step at 72°C for 30 s. A final extension time of 2 min at 72°C completed the reaction. Amplified products were run on an ABI3700 to separate fragments. The program GENEMARKER v. 1.9.1 (SoftGenetics, LLC) was used to determine allele sizes. All samples were scored by two different people and scores were compared on an online server [32] to detect inconsistencies and scoring errors. Loci that showed atypical profiles or low amplification were amplified a second time either in singleplex reactions or in multiplexes including fewer loci to increase amplification product. The analysis-ready dataset had a minimum of eight amplified loci per sample and showed no systematic dropout of specific loci.

One locus, BMS1788, showed a distinct mutational pattern. The locus is considered to have 1 bp alleles [18]. However, upon further investigation into allelic migration patterns and DNA sequencing, it became clear that even 1 bp allele binning does not capture the mutational complexity of this locus. This is because the repeat motif consists of GT/GC in caribou leading to minor differences in electrophoretic migration patterns depending on repeat motif combinations. Hence, 1 bp binning does underestimate the genetic variation at this locus and next-generation sequencing may be required to fully understand the allelic diversity in BMS1788.

Therefore, this locus was taken out of further analysis, although the phylogeographical patterning of the sequence variant alleles was described.

#### 2.2.3 Mitochondrial DNA control region sequencing

Control region data were partly taken from [3] and additional sequence data were generated for this study, including 31 new haplotypes (GenBank accession nos. KM016758–KM016788). MtDNA control region fragments were amplified and sequenced using the following primers [11]: l15394: 5′-AAT AGC CCC ACT ATC AGC ACC C-3′ and H15947: 5′-TAT GGC CCT GAA GTA AGA ACC AG-3′. PCR protocols and laboratory procedures used for this study were identical to the ones previously described [3]. Amplified PCR products were sequenced on an ABI3700 and checked manually by eye using BioEdit v. 7.2.0 [33]. Subsequently, alignments were done with the same program and mutated positions were manually checked another time to detect any potential remaining sequencing errors. In order to distinguish haplotypes, the program DnaSP v. 5 [34] was used with default settings. All newly detected haplotypes were sequenced to confirm the novel sequences.

#### 2.2.4 Statistical data analysis

We used the software Micro-Checker v. 2.2.3 [35] to assess potential genotyping errors, large allele dropout and the presence of null alleles in the microsatellite dataset. We used GenePop on the Web v. 4.2 [36] to calculate significant deviations from Hardy–Weinberg equilibrium (HWE) and linkage disequilibrium (LD) per locus and population (electronic supplementary material, S2.5 and S2.6). For HWE, we used a Markov chain method with 10 000 dememorization steps, 5 000 batches and 10 000 iterations each to estimate exact *p*-values for deficiency of heterozygotes and *F*_*IS*_ [37]. We used a log likelihood ratio statistics for LD. Finally, we used the program HP-Rare v. 1.1 [38,39] to calculate allelic richness and private allelic richness per population. Following identification of population clusters in STRUCTURE [40], we calculated genetic diversity estimates (i.e. expected and observed heterozygosity, number of alleles) with GenAlEx v. 6.5 [41] between STRUCTURE identified population clusters.

For microsatellites and mtDNA, we conducted an analysis of molecular variance (AMOVA) with Arlequin v. 3.5 [42] to test whether mtDNA supported the distinction of populations identified in STRUCTURE. We ran Arlequin with 10 000 permutations and calculated summary statistics (i.e. nucleotide and gene diversity) with the same program.

#### 2.2.5 STRUCTURE analysis

The Bayesian clustering program structure 2.3.4 [40] was used to assess the most likely number of population clusters (*K*) and to assign individuals to the inferred population clusters. We used an admixture model with correlated allele frequencies [43] and a burn-in of 1×10^6^ followed by 1×10^7^ permutations to test *K*=1 to *K*=15 with five iterations each to calculate *q*, the membership coefficient, representing proportional individual memberships to different inferred population clusters, thereby indicating if an individual showed admixture of two or more population clusters. We did not use prior location information to assist clustering of individuals. We ran the program on a high-performance computing cluster (SHARCNET—www.sharcnet.ca). Finally, we used the program STRUCTURE HARVESTER v. 0.6.93 [44] to summarize run statistics. To identify the most probable number of population clusters, we used the *ΔK* method [45]. We used the programs CLUMPP v. 1.1.2 [46] and DISTRUCT v. 1.1 [47] to average individual and population membership *q* values over the five iterations to retrieve highly reliable estimates of individual membership coefficients and to visualize results of the Bayesian assignment analysis. To identify admixed individuals between inferred population clusters in STRUCTURE, we applied a threshold *q*-value of more than or equal to 0.8 to assign an individual as belonging to a specific group and to assign individuals to distinct groups. Similarly, a *q*-value of less than 0.8 indicated an admixed individual. These or similar relaxed cut-off values have been widely used in the literature [48–50].

#### 2.2.6 Approximate Bayesian computation

ABC is a recently developed simulation method that avoids exact likelihood calculations by using summary statistics (i.e. values calculated from the data that capture the maximum amount of information in its simplest form [51]) and simulations to test competing demographic and evolutionary history models [51–53]. Briefly, a large number of datasets are simulated for a given evolutionary scenario. Subsequently, the simulated data are reduced to summary statistics and compared to observed summary statistics in the dataset. The distance between simulated and observed summary statistics determines if sampled parameters are accepted or rejected, thereby providing a measurement of fit for the evolutionary model investigated [51–53]. ABC has been widely applied to test competing evolutionary scenarios to detect past divergence events [51–53], recent and historical secondary contact events [53,54] and gene flow between populations [54]. Recently, the method has been used to explain the evolutionary history of species of interest to conservation managers and policy makers to inform conservation and management [54–58].

**Figure 2.**
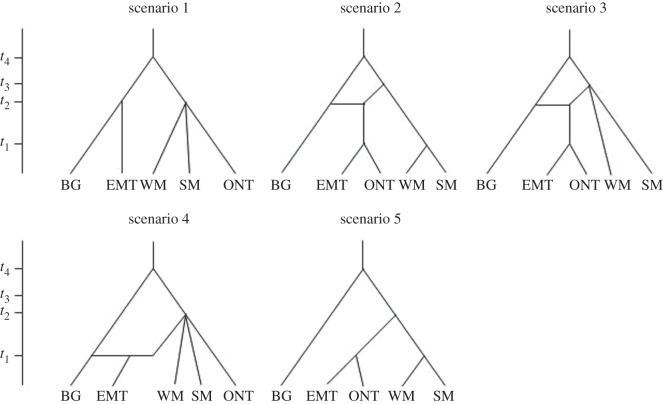
Evolutionary scenarios tested using DIYABC [21]. BG, barren-ground; EMT, eastern migratory ecotype; WM, western Manitoba; SM, southwestern Manitoba; ONT, Ontario and eastern Manitoba.

We used the software package DIYABC v. 2.0.4 [21] to test two major groups of models in order to identify the most supported evolutionary scenario [21,59]. The first group included divergence events without admixture (scenarios 1 and 5, figure 2). Two alternative scenarios are possible: (1) the eastern migratory ecotype descended from barren-ground caribou and there was little to no introgression of genetic material from woodland caribou (figure 2, scenario 1) or vice versa (figure 2, scenario 5). We first tested additional split scenarios (i.e. the eastern migratory ecotype could have diverged at different time points) separately to identify the most likely split models (electronic supplementary material, S2.11) that subsequently were included in the tests for admixture (figure 2). The second group of models included divergence with admixture (scenarios 2–4, figure 2). From these, we determined whether a specific woodland caribou group could be identified that mixed with barren-ground resulting in the eastern migratory ecotype and if a sub-specific admixture event resulted in an admixed population that pre-dated the evolution of the eastern migratory ecotype.

#### 2.2.7 Prior and conditions for approximate Bayesian computation analysis

The program jModeltest v. 2.1.4 [60] was used to identify the best suited substitution model and proportion of invariable sites. These parameters were used to set the mutation model in DIYABC. For microsatellites, we chose a stepwise mutation model with a mean mutation rate of 1×10^−5^ to 1×10^−3^. The merging of the boreal caribou groups was initially set to *t*_3_=1000–4000 generations, values that reflected the last glacial maximum when the three existing woodland caribou lineages (A1–A3) evolved. Similarly, the merging of the two sub-specific lineages was set to *t*_4_=10 000–60 000 generations based on estimates in other studies [14]. The admixture event was assumed to be the most recent event after formation of caribou groups and was set to *t*_2_=200–2000 generations. Finally, the subsequent divergence of the eastern migratory ecotype was assumed to be the youngest event with *t*_1_=10–1000 generations. Generation times of 4–7 years for caribou are reported in the literature [15,61–63] and we assumed that a generation time of 7 years was the most likely [61]. Thus, to estimate years, we multiplied the estimated generation times by 7. Finally, we used the following summary statistics: a) for within populations statistics: mean number of alleles, mean size variance of alleles, mean number of haplotypes, mean and variance of pairwise difference of sequences, mean and variance of number of the rarest nucleotide at segregating sites; b) for each population pair: mean number of alleles, mean size variance of alleles, *F*_ST_ (for both microsatellites and haplotypes), shared allele distance, classification index, number of haplotypes, and mean pairwise difference within and between populations.

The admixture event was set earlier in the tested models than the divergence into different boreal groups with the exception of scenario 4, which assumed that a population split from an ancient woodland caribou group and subsequently admixed with barren-ground. All boreal groups diverged from one most recent common ancestor because they belong to one phylogenetic group [3]. Similarly, the two sub-specific lineages (i.e. woodland and barren-ground caribou; figure 1) diverged from one most recent common ancestor and it was assumed that the subspecies split occurred earlier than the split into different populations.

We ran approximately 3 million simulations and compared scenarios by estimating posterior probabilities using the logistic regression [64]. For model comparison, the ‘logistic regression’ option in combination with a linear discriminant analysis [64] in DIYABC v. 2.0.4 was used. Briefly, the ‘logistic regression’ uses a polychotomic weighted logistic regression [52] on a pre-defined number of simulated datasets. Sample size for the logistic regression was 1% of the original number of simulated datasets (approx. 30 000 simulated datasets; electronic supplementary material, S2.8*a*–*c*). The assessment of the goodness-of-fit of a model parameter posterior combination was tested via the model-checking option in DIYABC v. 2.0.4 [21]. Runs were performed with default settings and for all three datasets (i.e. microsatellites, mtDNA and combined dataset; electronic supplementary material, S2.8*a*–*c*).

## 3. Results

### 3.1 Quality checks, Hardy–Weinberg equilibrium and linkage disequilibrium

In total, 1360 unique genotypes (67.1% females) were identified. Samples with at least eight out of 10 successfully scored loci were used in subsequent analysis (missing data in the microsatellite dataset was 2.9%). However, one locus (BMS1788) was taken out of further analysis because it showed mutational patterns that are inconsistent with 1 bp shifts.

Neither Micro-Checker tests nor tests for deviations from HWE and LD revealed any significant evidence that specific populations showed null alleles, deviated from HWE (9/333 cases significant after Bonferroni correction), or showed signs of LD (none significant after Bonferroni correction). Therefore, we retained nine loci for the subsequent analyses to examine phylogeographical structuring rather than delineate fine-scale local structure that might also exist but is beyond the scope of this work.

**Figure 3.**
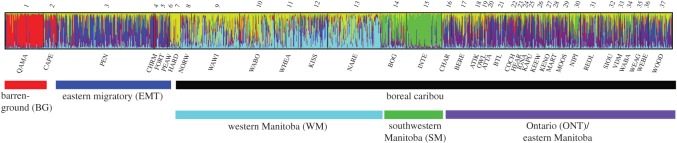
Bar plot (*K*=6) of the Bayesian clustering analysis for more than 1300 unique genotypes analysed at nine microsatellite loci using STRUCTURE v. 2.3.4 [40]. Population ranges are abbreviated as follows: QAMA, Qamanirjuaq; CAPE, Cape Churchill; PEN, Pen Island; CHRM, Cape Henrietta Maria; FORT, Fort Severn; PEAW, Peawanuck; HARD, Harding Lake; NORW, Norway House; WAWI, Wapisu-Wimapedi; WABO, Wabowden; WHEA, Wheadon; KISS, Kississing; NARE, Naosap-Reed; BOG, The Bog; INTE, North Interlake; CHAR, Charron Lake; BERE, Berens; ATIK, Atiko; OWL, Owl-Flintstone; ATTA, Attawapiskat; BTL, Big Trout Lake; COCH, Cochrane; HEAR, Hearst; IGNA, Ignace; KAPU, Kapukasing; KEEW, Keewaywin; KENO, Kenogami; MART, Marten Falls; MOOS, Moosonee; NIPI, Nipigon; REDL, Red Lake; SIOU, Sioux Lookout; VDM, Victor Diamond Mine; WABA, Wabakimi; WEAG, Weagamow; WEBE, Webequie; WOOD, Woodland Caribou Provincial Park.

**Figure 4.**
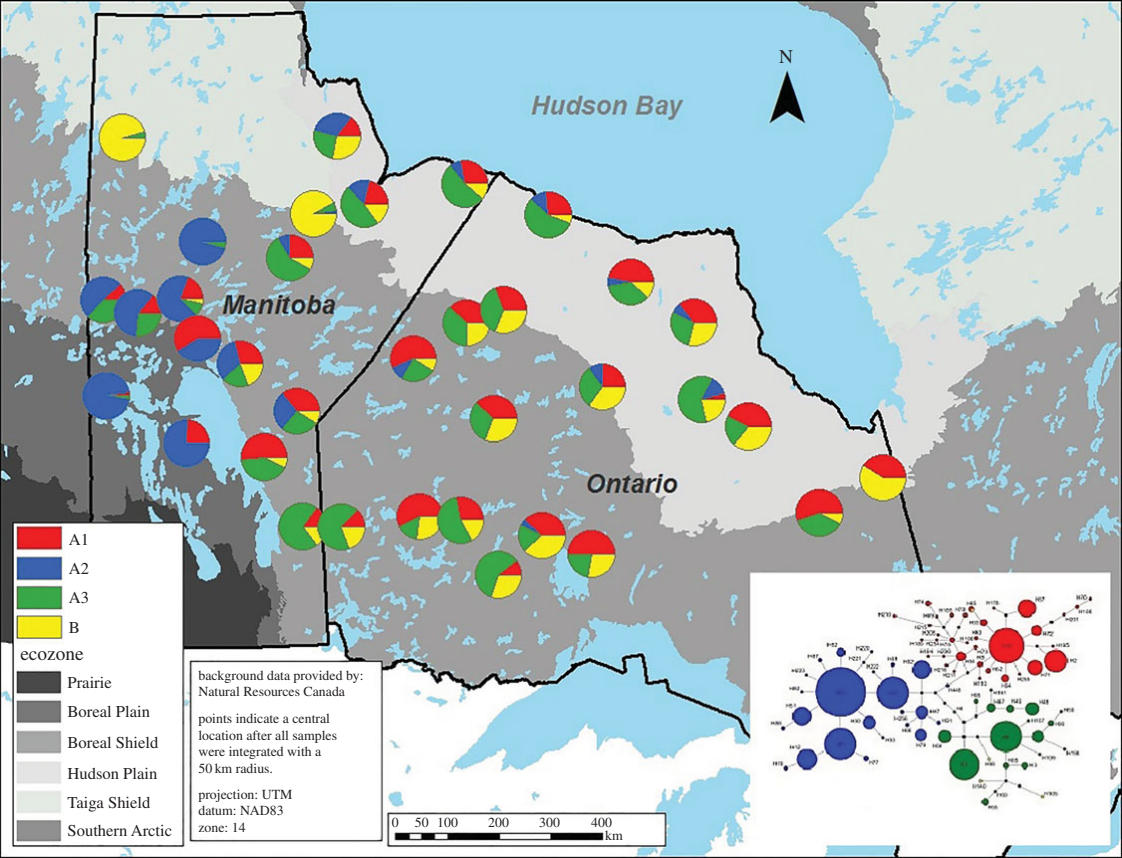
Geographical distribution of haplogroup diversity. Each pie chart represents the proportion of haplotypes belonging to the four identified haplogroups (*sensu* [3]) in a given population range. Yellow, haplogroup B; red, haplogroup A1; blue, haplogroup A2; green, haplogroup A3.

Analysis of nine microsatellite loci and 401 bp of the mtDNA control region showed distinctive genetic patterns. The STRUCTURE analysis identified two levels of population genetic structure in the dataset (figure 3). The first level, at *K*=2, corresponded to the subspecies level distinguishing barren-ground from all other populations (electronic supplementary material, figures S2.2 and S2.3). The second level, at *K*=6 (figure 3; electronic supplementary material, figure S2.4*a*,*b*), revealed population genetic structure at the ecotype and within-ecotype level, identifying barren-ground, the eastern migratory ecotype, including the Pen Island herd and coastal region in Ontario, as structured groups. Further, the boreal caribou ecotype consisted of four populations (figure 3; and electronic supplementary material, figures S2.4*a*,*b*): (1) and (2) two populations in western Manitoba, (3) one population in southwestern Manitoba and (4) one population including eastern Manitoba and Ontario mainland. For further analysis, the two populations found in western Manitoba were combined because only a single sampling site with fairly low sampling size (*N*=23) showed high membership coefficients for that cluster with additional individuals being highly admixed. It is currently unknown whether that cluster is biologically meaningful and the pattern requires further investigation with more local sampling. Since the focus here was to understand the evolutionary origin of the eastern migratory ecotype, western Manitoba was considered one population with potential substructure. All subsequent analyses were carried out with the five major groups identified in STRUCTURE. Further, a population mean *q* bar plot (electronic supplementary material, figure S2.4*a*) revealed that individuals of the eastern migratory ecotype had higher average membership assignments to their focal population. Likewise, Ontario/eastern Manitoba showed a different genetic signature including higher average membership coefficients to a different population cluster. Hence, the eastern migratory ecotype and the Ontario/eastern Manitoba population were treated as different populations in succeeding analyses. Additionally, a sorted *q* bar plot (electronic supplementary material, figure 2.4*b*) confirmed the presence of highly assigned individuals to each of the identified populations indicating that, albeit high admixture levels in Ontario, two distinct clusters exist there (i.e. eastern migratory ecotype and Ontario/eastern Manitoba) (figure 4).

Lastly, the phylogeographical pattern in BMS1788 is consistent with results in that a preliminary assessment of allele variation supports a phylogeographical pattern separating western Manitoba and Ontario. An AMOVA on microsatellite data revealed lower differentiation values that nevertheless were highly significant; therefore, supporting the results found in the STRUCTURE analysis.

Since barren-ground (B haplotypes) and woodland caribou (A haplotypes) clearly differed in mtDNA control region sequences [3,12,13], it was possible to detect differential introgression across the two provinces with the highest B introgression levels found in eastern Manitoba/Ontario. In western and southwestern Manitoba, very low proportions of B haplotypes were found (table 1). Out of 902 boreal caribou in Ontario/eastern Manitoba, 96 (10.6%) carried eight different B haplotypes in common with the eastern migratory ecotype but not found in barren-ground. This may reflect limited sampling of barren-ground caribou; however, recent introgression would likely reflect common barren-ground haplotypes.

**Table 1.** Summary of haplotype genetic diversity. Number of haplotypes (*N*), number of haplotypes from subhaplogroup A1 (*N* A1), A2 (*N* A2) and A3 (*N* A3). Further, number and percentage of A haplotypes (*N* (A) and A (%)) and B haplotypes (*N* (B) and B (%)) are given. Finally, nucleotide diversity (*π*) and gene diversity (gene div) plus respective standard deviations are shown.

group	*N*	*N* A1	A1 (%)	*N* A2	A2 (%)	*N* A3	A3 (%)	*N* (A)	A (%)	*N* (B)	B (%)	*π*	s.d.	gene div	s.d.
QAMA	76	0	0	0	0	5	6.6	5	6.6	71	93.4	0.02	0.01	0.98	0.01
EMT	234	61	26.1	26	11.1	116	49.6	203	86.8	31	13.2	0.02	0.01	0.9	0.01
WM	339	60	17.7	204	60.2	67	19.8	331	97.6	8	2.4	0.01	0.01	0.77	0.02
SM	108	18	16.7	89	82.4	1	0.9	108	100	0	0	0.01	0.01	0.82	0.01
ONT	455	162	35.6	14	3.1	191	42	367	80.7	88	19.3	0.02	0.01	0.93	0.01

A hierarchical AMOVA (mtDNA) revealed that approximately 22% of the genetic variation was found between the STRUCTURE groups indicating clear genetic structure (tables 2 and 3). Within woodland caribou, all populations were significantly differentiated from each other. Within the boreal caribou ecotype, western and southwestern Manitoba had a high haplogroup A2 proportion, whereas Ontario/eastern Manitoba had more haplotypes from haplogroups A1 and A3. Similarly, the eastern migratory ecotype also had a high proportion of A1 and A3 haplotypes in addition to barren-ground B haplotypes. To conclude, the contemporary spatial haplogroup distribution seen in this ecotype supports the secondary contact of divergent lineages in northern Manitoba.

**Table 2.** AMOVA based on haplotype and microsatellite data and the five identified groups by STRUCTURE (QAMA, EMT, WM, SM, ONT, respectively). *V*_a_—the between STRUCTURE groups component of variance, *V*_b_—the among-population component of variance, and *V*_c_—the within-population component of variance. *F*_ST_—the variance within populations relative to the total variance, *F*_SC_—the variance among populations within groups, and *F*_CT_—the variance among groups relative to the total variance.

source of variation	d.f.	variance components	% variation	*F*	*p*
mitochondrial DNA					
among groups	4	1.03 *V*_a_	21.9	*F*_CT_=0.22	0.0000
among populations within groups	31	0.35 *V*_b_	7.5	*F*_SC_=0.1	0.0000
within populations	1176	3.32 *V*_c_	70.7	*F*_ST_=0.29	0.0000
microsatellite data					
among groups	4	0.06 *V*_a_	1.7	*F*_CT_=0.02	0.0000
among populations within groups	31	0.1 *V*_b_	2.8	*F*_SC_=0.03	0.0000
within populations	2630	3.29 *V*_c_	95.5	*F*_ST_=0.04	0.0005

**Table 3.** (*a*) Pairwise *φ*_ST_ values based on mitochondrial DNA for groups identified in STRUCTURE (below diagonal) and pairwise *p*-values (above diagonal). (*b*) Pairwise *F*_ST_ values based on microsatellites for five major groups identified in STRUCTURE (below diagonal) and pairwise *p*-values (above diagonal).

	QAMA	EMT	WM	SM	ONT
(*a*)					
QAMA	—	0.000	0.000	0.000	0.000
EMT	0.41	—	0.000	0.000	0.000
WM	0.59	0.18	—	0.000	0.000
SM	0.65	0.31	0.08	—	0.000
ONT	0.33	0.03	0.21	0.31	—
(*b*)					
QAMA	—	0.000	0.000	0.000	0.000
EMT	0.07	—	0.000	0.000	0.000
WM	0.06	0.02	—	0.000	0.000
SM	0.08	0.04	0.04	—	0.000
ONT	0.07	0.01	0.01	0.03	—

ABC (i.e. microsatellite loci, mtDNA haplotypes and the combined dataset) identified scenario 3 as the most likely evolutionary scenario based on posterior probability values, credible intervals (table 4) and logistic regression (electronic supplementary material, S2.8). Scenario 3 suggests that the eastern migratory ecotype split from a lineage having experienced introgression from barren-ground caribou estimated to be approximately 6804 years before present (ybp; CI: 1680–17 010 ybp; combined dataset) versus approximately 6055 ybp (CI: 1309–16 870 ybp; microsatellites) and approximately 14 000 ybp (CI: 3199–25 620; mtDNA). Similarly, the divergence of the eastern migratory ecotype was estimated to be approximately 2982 ybp (CI: 591–6083 ybp; combined dataset) versus 4389 ybp (CI: 903–6713 ybp; microsatellites) and approximately 973 ybp (CI: 95.2–4739 ybp; mtDNA; electronic supplementary material, S2.10*a*–*c*). As would be expected for the rates of change at each type of loci and the time frame under investigation, these results suggest that mtDNA is more reliable in estimating the more ancient admixture event, whereas the microsatellite data are superior in estimating the more recent divergence event (electronic supplementary material, table S2.10*a*–*c*). Since mtDNA has a lower mutation rate than microsatellites, haplotype resolution may simply be insufficient to fully capture the divergence of eastern migratory and Ontario/eastern Manitoba boreal caribou, and therefore microsatellite data are likely more suitable for the analysis of the more recent divergence event. Cornuet *et al.* [64] pointed out that microsatellites alone performed poorly in estimating ancient divergence events, likely due to their unique characteristics (i.e. high mutation rates in combination with allele size homoplasy). Taking these considerations into account, the eastern migratory and Ontario/eastern Manitoba boreal caribou populations split most likely between 2982 ybp (combined dataset) and 4389 ybp (microsatellite dataset). The introgression event occurred between approximately 6804 ybp (combined dataset) and approximately 14 000 ybp (mtDNA dataset) with the latter probably being more accurate.

**Table 4.** Posterior probability and credible interval [CI] for the three ABC runs (i.e. microsatellite, mtDNA and combined dataset). In all three cases, scenario 3 is selected as the most supported model.

	scenario 1	scenario 2	scenario 3	scenario 4	scenario 5
microsatellite dataset	0.000 [0.000–0.398]	0.000 [0.000–0.400]	1.000 [0.999–1.000]	0.000 [0.000–0.400]	0.000 [0.000–0.400]
mtDNA dataset	0.003 [0.000–0.123]	0.044 [0.000–0.162]	0.879 [0.862–0.895]	0.042 [0.000–0.159]	0.033 [0.000–0.160]
combined dataset	0.000 [0.000–0.178]	0.036 [0.000–0.210]	0.945 [0.935–0.955]	0.001 [0.000–0.178]	0.019 [0.000–0.200]

## 4. Discussion

Increased understanding of the evolutionary processes that lead to contemporary spatial genetic partitioning is critical for delineating units for conservation assessment and subsequent recovery plans and may enhance our ability to protect important adaptive potential of species at risk [1,65,66]. In this study, we identified five differentiated caribou groups in Manitoba and Ontario that showed different mtDNA introgression levels from barren-ground caribou. ABC analyses supported divergence of an eastern migratory ecotype from a woodland caribou lineage that had experienced ancient introgression from barren-ground caribou. Only low levels of recent sub-specific introgression (figure 2) could be detected. Our results suggest that the evolution of the eastern migratory ecotype dated to shortly after the retreat of the Laurentide ice sheet but was not directly coupled to the introgression event. This links the eastern migratory ecotype origin to the post-glacial emergence of the land and establishment of vegetation in the Hudson Bay coastal areas approximately 7000 years ago [67,68].

Under this scenario, barren-ground caribou likely migrated along the retreating ice sheet allowing for lineage mixing in northern Ontario and Manitoba. The eastern migratory ecotype diverged from this ancient introgressed population, leaving residual barren-ground-specific haplotypes in both the eastern migratory ecotype and Ontario/eastern Manitoba boreal caribou population. The lack of B haplotypes in western and southwestern Manitoba might be best explained by later arriving western (i.e. A2 lineage) caribou populations that migrated eastwards and consequently did not readily interbreed with barren-ground. The occurrence of B haplotypes in eastern migratory and the boreal ecotypes in Ontario and eastern Manitoba is possibly the result of the ancient introgression event identified in the ABC analysis. Alternatively, gene flow between the eastern migratory ecotype and boreal caribou populations led to distributions of a few B haplotypes.

In western Canada, several studies [12,13] detected relatively high levels of mtDNA introgression which was hypothetically facilitated by enduring genetic interchange because of an ice-free corridor along the eastern side of the Rocky Mountains that formed approximately 12 000–14 000 years ago [67,68]. However, similar ice-free corridors may also have existed 24 000–40 000 years ago along the Pacific coast [69] potentially facilitating more ancient introgression. The diverse source populations and regional and temporal variations in deglaciation have likely led to differential timing of secondary contacts between northern and southern evolved lineages across Canada and therefore various evolutionary outcomes. The hypothesized long-term contact and reciprocal introgression between northern and southern caribou potentially led to the evolution of ecotypes (i.e. northern and southern mountain).

McDevitt *et al*. [12] also suggested that the admixture of A and B lineages resulted in the transfer of genetic material increasing migratory behaviour in woodland caribou. However, mtDNA introgression into two different caribou ecotypes (i.e. eastern migratory and sedentary boreal) does not support the hypothesis that (ancient) barren-ground introgression directly relates to increased migratory behaviour. While nuclear microsatellites support the divergence of the eastern migratory ecotype independent of the ancestral introgression event, we cannot exclude that low levels of gene introgression may offer a predisposition for adaptation of the migratory behaviour. We are also inferring that the migratory behaviour of the eastern migratory ecotype would have been derived from the introgressed woodland lineage as a result of colonization following Holocene vegetation changes along the emerging Hudson Bay coast. However, we cannot reject that migratory behaviour pre-existed in the woodland lineage prior to the introgression event. Certainly, other factors like behavioural plasticity determined by ecological conditions (e.g. [70]) may be underlying migratory behaviour in caribou.

Migratory behaviour has been shown to increase fitness through exposure to seasonal changes in predator avoidance [71,72] and resource availability [70,72]. Thus, migration gives barren-ground caribou greater protection from predators and access to high quality plant growth during spring green-up (compared to boreal animals) that may ultimately result in higher growth rates and larger population sizes. The situation in Quebec is less well studied with mtDNA, but preliminary results suggest that at least low levels of mtDNA introgression may be present in some regions [11,14]. To what extent the eastern migratory ecotype in Quebec has a close relationship with those surveyed in this study in Ontario and Manitoba remains to be tested. Although extensive recent admixture between caribou subspecies is rejected in the formation of the eastern migratory ecotype in Manitoba and Ontario, mtDNA introgression points to the possibility that adaptive alleles at nuclear genes may also have introgressed into woodland caribou, and even at low levels, these alleles may subsequently manifest in certain environments or ecozones [5,73,74]. The impact of relative levels of differential introgression is becoming increasingly studied [75].

Regardless of the source of migratory behaviour, lineage-specific diversity is important, both biologically and for conservation ranking because genetically, different populations in widespread species often follow independent evolutionary trajectories and therefore may respond differently to new or changing environments [74,75]. The consideration of relationships of sub-specific lineages and the role of secondary contact zones across Canada in ecotype formation is warranted in conservation and management strategies. Likewise, a better understanding of the impact of post-Pleistocene and Early Holocene dynamics following glacial retreat and colonization to emerging ecozones on the evolution and adaptation of conservation units below the subspecies level is necessary for the implementation of conservation strategies that capture independent evolutionary trajectories and adaptive genetic variation in caribou and other species.

The maintenance of significant phylogeographical structure found in this study also points to the potential importance of additional environmental factors and landscape features (e.g. ecozones, environmental gradients and post-Pleistocene vegetation history) in shaping of genetic diversity. Since environmental factors are likely to be diverse across the distribution range of widespread species, locally adapted populations will be spatially distributed as well. It has been proposed [76] that protecting and connecting populations along climatic gradients may trigger adaptive evolution, thereby preserving and potentially increasing evolutionary potential. The integration of evolutionary and functional genetics into conservation and management practices will therefore be essential to detect, preserve and connect genetic variation across a species’ distribution range and to identify key areas that potentially provide neutral and functional genetic variation to respond to rapid climate change. Finally, the aspects discussed here point to the need of implementing nationwide and flexible conservation approaches for widespread species.

## Supplementary Material

Electronic Supplementary Material

## Data accessibility

The microsatellite dataset has been deposited with Dryad (Dryad Digital Repository:). GenBank accession numbers for new mtDNA control region haplotypes: KM016758–KM016788.
