# Supplementary material for: The eastern migratory caribou: the role of genetic introgression in ecotype evolution
Source: R Soc Open Sci. 2016 Feb 3;3(2):150469. doi: 10.1098/rsos.150469 (PMC4785971; doi:10.1098/rsos.150469)
Supplement: Electronic Supplementary Material [file rsos150469supp1.docx]

**Electronic Supplementary Material**

**The eastern migratory caribou: the role of genetic introgression in ecotype evolution**

C. F. C. Klütsch, M. Manseau, V. Trim, J. Polfus, P. J. Wilson

**Table of contents Pages**

**S1. Distribution ranges and life history notes** 5

**Figure S1.1.** Caribou sampling locations in Manitoba and Ontario, Canada. 6

**S2. Laboratory procedures for DNA extraction, microsatellite genotyping, and mitochondrial DNA sequencing** 6 - 7

**S3. Microsatellite amplification and genotyping** 7 - 8

**S4. Mitochondrial DNA control region sequencing** 8 - 9

**S5. Statistical data analysis** 9 - 10

**S6. Background STRUCTURE analysis** 10

**S7. Background Approximate Bayesian Computation (ABC)** 11 - 12

**S8. Additional Information for Results** 13

**S8.1. Details for quality checks, Hardy-Weinberg equilibrium (HWE),**

**and linkage disequilibrium (LD)** 13

**Figure S8.2.** Mean likelihood for each K plus standard deviation as retrieved

from STRUCTURE Harvester version 0.6.93 [1]. 14

**Figure S8.3.** Most likely number of population clusters (K = 2 and K = 6)

identified by the Evanno method [2] using STRUCTURE Harvester

version 0.6.93 [1]. K = 6 consistently retrieved five major groups plus a

subtle substructure in western Manitoba. 15

**Figure S8.4.**  Bar plot of the Bayesian clustering analysis for > 1300 unique genotypes

analysed at 9 microsatellite loci using STRUCTURE 2.3.4 [3]. Population ranges are

abbreviated as follows: QAMA = Qamanirjuaq, CAPE = Cape Churchill,

PEN = Pen Island, CHRM = Cape Henrietta Maria, FORT = Fort Severn,

PEAW = Peawanuck, HARD = Harding Lake, NORW = Norway House,

WAWI = Wapisu-Wimapedi, WABO = Wabowden, WHEA = Wheadon,

KISS = Kississing, NARE = Naosap-Reed, BOG = The Bog, INTE = North Interlake,

CHAR = Charron Lake, BERE = Berens, ATIK = Atiko, OWL = Owl-Flintstone,

ATTA = Attawapiskat, BTL = Big Trout Lake, COCH = Cochrane, HEAR = Hearst,

IGNA = Ignace, KAPU = Kapukasing, KEEW = Keewaywin, KENO = Kenogami,

MART = Marten Falls, MOOS = Moosonee, NIPI = Nipigon, REDL = Red Lake,

SIOU = Sioux Lookout, VDM – Victor Diamond Mine, WABA = Wabakimi,

WEAG = Weagamow, WEBE = Webequie,

WOOD = Woodland Caribou Protection Park. 16

**Table S8.5.** Summary of genetic diversity estimates per microsatellite.

Allelic range, number of alleles (N_A_), expected (H_E_) and observed

heterozygosity (H_O_), F_IS_ estimates, and standard errors (SE) for each of

the estimates is given. Original references for loci are given. 17

**S8.6.** Principal Component Analysis (PCA) pre-evaluation plot generated by

DIYABC version 2.0.4 [4,5] showing a representative set (10,000 simulated

data set per scenario) of simulated data sets from the reference table

(small dots) and the observed data (large yellow dot). As displayed, the plot

shows that the observed data falls into a cloud of simulated data sets,

indicating that it is possible to produce a subset of summary statistics

close to the observed summary statistics with the proposed scenarios in

combination with the chosen prior distributions. 18 - 19

**S8.7.** Comparison of scenarios ranking the posterior probabilities for each

tested scenario. 20 – 22

**S8.8. Time estimates – Approximate Bayesian computation (ABC)** 23 - 24

**S9. Preliminary results for Cape Churchill herd** 25

**S10. A note on hybrid swarms** 26

**S11. A note on the metapopulation model** 26 - 27

**S12. References for Supplementary Material** 27 - 37

**S1. Distribution ranges and life history notes**

Boreal caribou have a large geographical distribution spanning from Labrador/Newfoundland and central Quebec to the northeast corner of the Yukon Territory and are often ecologically characterized as sedentary (sedentary ecotype *sensu* [6, 7-9], because they do not display extensive migrations or substantial overlap between seasonal ranges [10-13]. Other characteristics of this ecotype include low densities, a tendency for females to disperse across the landscape at calving to avoid predators [14] and little variance in population sizes if undisturbed. In contrast, eastern migratory caribou [12], which inhabit the open-tundra and boreal regions of northern Labrador, Québec, Ontario and Manitoba, show aggregated calving behaviour that is characteristic of barren-ground caribou and undertake long-distance annual migrations to calving grounds. The eastern migratory caribou ecotype encompasses a minimum of three populations (specifically George River, Leaf River, and Pen Island) with a possible fourth (Cape Churchill). Population sizes can vary significantly with 100-fold fluctuations reported [12, 15]. Similarly, the population size of barren-ground caribou herds can be very large; for example, the Qamanirjuaq herd size ranged between ~350,000 - ~500,000 individuals between the 1980s and 2008 [16]. The core distribution range of barren-ground caribou extends from the subarctic tundra near the Mackenzie Delta in the Northwest Territories to the eastern coast of mainland Nunavut, and south into northern Saskatchewan and Manitoba [12, 17]. Population ranges of two barren-ground herds in the Yukon, the Porcupine and Forty-Mile herds, extend into Alaska where similarly large herds of migratory tundra caribou occur across the tundra ecosystem [12].

**Figure S1.1.** Caribou sampling locations in Manitoba and Ontario, Canada.

**
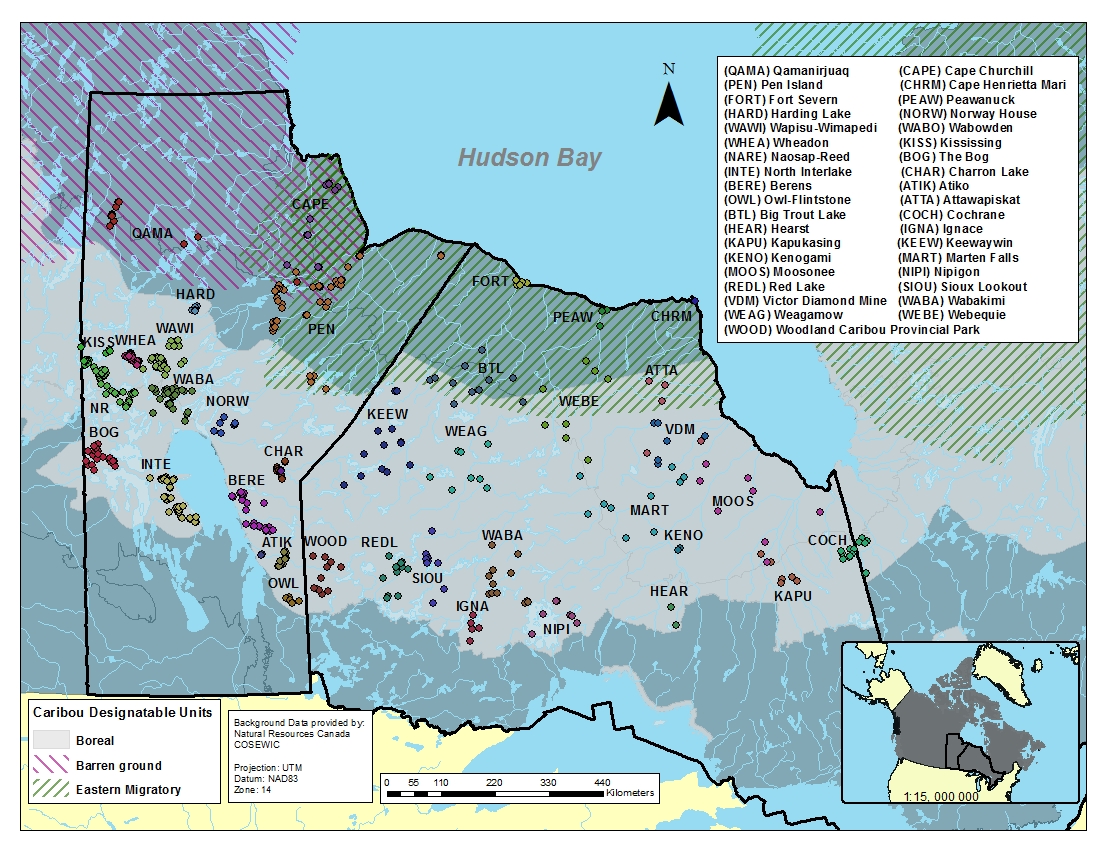
**

**S2. Laboratory procedures for DNA extraction, microsatellite genotyping, and mitochondrial DNA sequencing**

DNA was extracted from fecal pellets using the protocol outlined in [18, 19]. Briefly, a sterile cotton-tipped applicator (Puritan) was used to swab fecal pellets targeting the outer mucous layer in order to obtain epithelial cells for DNA extraction. Subsequently, swabs were placed into 300 μl of 1x lysis buffer. Then, a two-step digestion was carried out using 20 units of proteinase K (Roche Applied Science) in a first incubation step of 2h at 65°C to be followed by a second digestion step using an additional 20 units of proteinase K and an incubation period of 12 hours at 37°C. Next, DNA extraction was carried out using a DNeasy Blood and Tissue Kit (QIAGEN) following the manufacturer’s protocol. Finally, samples were eluted in preheated (~70°C) 65.0 μl of 0.1 M TE buffer and stored at -20°C until further processing.

**S3. Microsatellite amplification and genotyping**

Extracted DNA samples were amplified at ten microsatellite loci. Of these, six loci (Rt5, Rt6, Rt7, Rt9, Rt24, Rt30) were taken from [20] with the reverse 5’ taken from [21] for Rt5, two markers were taken from [22] (BM848 and BM888), and one marker each from [23] (BMS1788) and [22,24] (Map2C). Amplification of all loci was conducted in three multiplex reactions containing the following primer combinations: multiplex 1: Rt6, Rt9, Map2C; multiplex 2: Rt5, Rt30; multiplex 3: BMS1788 and Rt7; and four singleplexes for BM888, BM848, Rt24, and sex determination [18, 19, 25]. Reactions were performed in a 7 μl volume containing: 1x PCR buffer; 2.0 mM MgCl; 0.2 μg/ml of BSA; 0.4 μM of each primer pair (forward and reverse) according to the abovementioned multiplex combinations; 0.2 μM of each dinucleotide triphosphate; 0.5 unit of *Taq* polymerase (Invitrogen Life Technologies) and 5 ng of DNA template. The thermocycling protocol included the following steps: a denaturation step at 95°C for 10 min, followed by 30 cycles of 94°C for 30 s, an annealing step for 60 s at 56°C for multiplex 1 (58°C for multiplex 3 and 60°C for multiplex 2 and singleplexes), and 72°C for 1 min. A final extension time of 65°C for 15 min completed the reaction. For the sexing reaction, the thermocycling protocol included a denaturation step at 94°C for 5 min, followed by 30 cycles of 94°C for 30 s, an annealing step for 30 s at 56°C, and an extension step at 72°C for 30 sec. A final extension time of 2 min at 72°C completed the reaction. Amplified products were run on an ABI3700 to separate fragments. The program GENEMARKER v. 1.9.1 (SoftGenetics, LLC) was used to determine allele sizes. All samples were scored by two different people and scores were compared on an online server [26] to detect inconsistencies and scoring errors. Loci that showed atypical profiles or low amplification were amplified a second time either in singleplex reactions or in multiplexes including fewer loci to increase amplification product. The analysis-ready data set had a minimum of eight amplified loci per sample and showed no systematic dropout of specific loci.

One locus, BMS1788, showed a distinct mutational pattern. The locus is considered to have 1 bp alleles [27]. However, upon further investigation into allelic migration patterns and DNA sequencing, it became clear that even 1 bp allele binning does not capture the mutational complexity of this locus. This is because the repeat motif consists of GT/GC in caribou leading to minor differences in electrophoretic migration patterns depending on repeat motif combinations. Hence, 1 bp binning does underestimate the genetic variation at this locus and next-generation sequencing may be required to fully understand the allelic diversity in BMS1788.

Therefore, this locus was taken out of further STRUCTURE analysis, although the phylogeographical patterning of the sequence variant alleles was described.

**S4. Mitochondrial DNA control region sequencing**

Control region data were partly taken from [28] and additional sequence data were generated for the current study, including 31 new haplotypes (GenBank accession numbers XXX - XXX). MtDNA control region fragments were amplified and sequenced using the following primers [29]: L15394: 5’ - AAT AGC CCC ACT ATC AGC ACC C- 3’ and H15947: 5’ - TAT GGC CCT GAA GTA AGA ACC AG – 3’. PCR protocols and lab procedures used for the current study were identical to the ones previously described [28]. Amplified PCR products were sequenced on an ABI3700 and checked manually by eye using BioEdit 7.2.0 [30]. Subsequently, alignments were done with the same program and mutated positions were manually checked another time to detect any potential remaining sequencing errors. In order to distinguish haplotypes, the program DnaSP v5 [31] was used with default settings. All newly detected haplotypes were sequenced to confirm the novel sequences.

**S5. Statistical data analysis**

We used the software Micro-checker v. 2.2.3 [32] to assess potential genotyping errors, large allele dropout, and the presence of null alleles in the microsatellite data set. We used GenePop on the Web v.4.2 [33] to calculate significant deviations from Hardy-Weinberg equilibrium (HWE) and linkage disequilibrium (LD) per locus and population. For HWE, we used a Markov chain method with 10,000 dememorization steps, 5,000 batches, and 10,000 iterations each to estimate exact *P*-values for deficiency of heterozygotes and F_IS_ [34]. We used a log likelihood ratio statistics for linkage disequilibrium. Finally, we used the program HP-Rare ver 1.1 [35, 36] to calculate allelic richness and private allelic richness per population.

Following identification of population clusters in STRUCTURE [3], we calculated genetic diversity estimates (i.e., expected and observed heterozygosity, number of alleles) with GenAlEx v. 6.5 [37] between STRUCTURE identified population clusters.

For mitochondrial DNA, we conducted an AMOVA with Arlequin version 3.5 [38] to test whether mtDNA supported the distinction of populations identified in STRUCTURE. We ran Arlequin with 10,000 permutations and calculated summary statistics (i.e., nucleotide and gene diversity) with the same program.

**S6. Background STRUCTURE analysis**

We used an admixture model with correlated allele frequencies [39] and a burn-in of 1 x 10^6^ followed by 1 x 10^7^ permutations to test K = 1 to K = 15 with five iterations each to calculate *q*, the membership coefficient, representing proportional individual memberships to different inferred population clusters, thereby indicating if an individual showed admixture of two or more population clusters. We did not use prior location information to assist clustering of individuals. We ran the program on a high-performance computing cluster (SHARCNET - www.sharcnet.ca). Finally, we used the program STRUCTURE HARVESTER v. 0.6.93 [1] to summarize run statistics. To identify the most probable number of population clusters, we used the ∆K method [2]. We used the programs CLUMPP v.1.1.2 [40] and DISTRUCT v.1.1 [41] to average individual and population membership *q* values over the five iterations to retrieve highly reliable estimates of individual membership coefficients and to visualize results of the Bayesian assignment analysis. To identify admixed individuals between inferred population clusters in STRUCTURE, we applied a threshold *q* value of ≥0.8 to assign an individual as belonging to a specific group and to assign individuals to distinct groups. Similarly, a *q* value ≤ 0.8 indicated an admixed individual. These or similar relaxed cut-off values have been widely used in the literature [42-44].

**S7. Background Approximate Bayesian Computation (ABC)**

Approximate Bayesian Computation (ABC) is a recently developed simulation method that avoids exact likelihood calculations by using summary statistics (i.e., values calculated from the data that capture the maximum amount of information in its simplest form [45]) and simulations to test competing demographic and evolutionary history models [4,5,45,46]. Briefly, a large number of data sets are simulated for a given evolutionary scenario. Subsequently, the simulated data is reduced to summary statistics and compared to observed summary statistics in the data set. The distance between simulated and observed summary statistics determines if sampled parameters are accepted or rejected, thereby providing a measurement of fit for the evolutionary model investigated [4,5,45,46]. ABC has been widely applied to test competing evolutionary scenarios to detect past divergence events [45,46], recent and historical secondary contact events [46,47], and gene flow between populations [47]. Recently, the method has been used to explain the evolutionary history of species of interest to conservation managers and policy makers to inform conservation and management [47-51].

**Prior and conditions for ABC analysis**

The program jModeltest version 2.1.4 [52] was used to identify the best suited substitution model and proportion of invariable sites. These parameters were used to set the mutation model in DIYABC. For microsatellites, we chose a stepwise mutation model with a mean mutation rate of 1x 10^-5^ to 1 x 10^-3^. The merging of the boreal caribou groups was initially set to t_3_ = 1000 – 4000 generations, values that reflected the last glacial maximum when the three existing woodland caribou lineages (A1-A3) evolved. Similarly, the merging of the two subspecific lineages was set to t_4_ = 10,000 – 60,000 generations based on estimates in other studies [53]. The admixture event was assumed to be the most recent event after formation of caribou groups and was set to t_2_ = 200 – 2000 generations. Finally, the subsequent divergence of the eastern migratory ecotype was assumed to be the youngest event with t_1_ = 10 – 1000 generations. Generation times of 4 – 7 years for caribou are reported in the literature [11, 54-57] and we assumed that a generation time of 7 years was the most likely [54]. Thus, to estimate years, we multiplied the estimated generation times by 7. Finally, we used the following summary statistics: a) for within populations statistics: mean number of alleles, mean size variance of alleles, mean number of haplotypes, mean and variance of pairwise difference of sequences, mean and variance of number of the rarest nucleotide at segregating sites; b) for each population pair: mean number of alleles, mean size variance of alleles, F_ST_ (for both microsatellites and haplotypes), shared allele distance, classification index, number of haplotypes, and mean pairwise difference within and between populations.

We ran approximately three million simulations and compared scenarios by estimating posterior probabilities using the logistic regression [58].

For model comparison, the ‘logistic regression’ option in combination with a linear discriminant analysis [4] in DIYABC version 2.0.4 was used. Briefly, the ‘logistic regression’ uses a polychotomic weighted logistic regression [5] on a pre-defined number of simulated data sets. As seen below (Fig. S8.7a - c), all three ABC runs (i.e., microsatellite data set and mtDNA separately as well as the combined data set) identified scenario 3 (i.e., admixture of woodland and barren-ground gave rise to an admixed population that subsequently split into eastern migratory and a boreal caribou population in Ontario) to be the best supported scenario in this comparison.

**S8. Additional Information for Results**

**S8.1. Details for quality checks, Hardy-Weinberg equilibrium (HWE), and linkage disequilibrium (LD)**

In an initial Micro-checker analysis, none of the loci showed signals of null alleles in more than 50% of the 37 sampling locations. However, we found that one locus (RT30) displayed signals of null alleles in 14/37 sampling sites, respectively. This is consistent with the test results for HWE, in which 46 out of 333 possible tests were significant at the 0.05 level. After Bonferroni correction (0.05/333 = 0.00015) only 9 tests (all RT30) remained significant. Out of 1194 pairwise comparisons for linkage disequilibrium, 72 were significant at the 0.05 level. However, after Bonferroni correction, none of the pairwise loci comparisons for linkage disequilibrium remained significant.

**Figure S8.2.** Mean likelihood for each K plus standard deviation as retrieved from STRUCTURE Harvester version 0.6.93 [1].


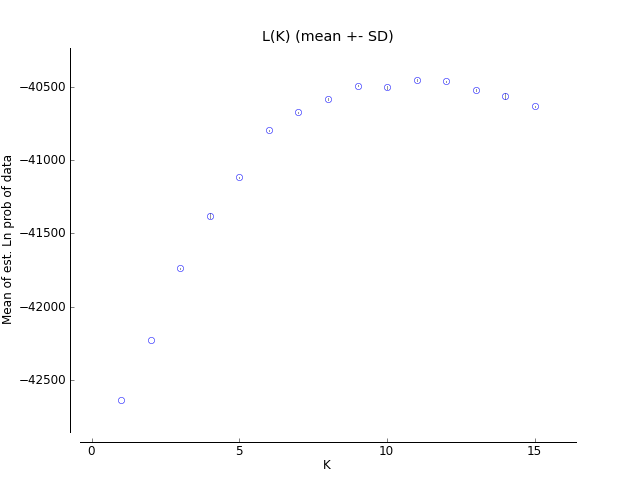


**Figure S8.3.** Most likely number of population clusters (K = 2 and K = 6) identified by

the Evanno method [2] using STRUCTURE Harvester version 0.6.93 [1]. K = 6 consistently retrieved five major groups plus a subtle substructure in western Manitoba.


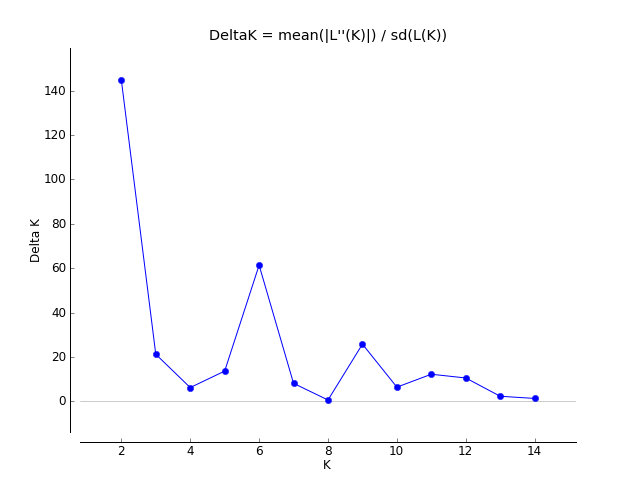


**Figure S8.4.** Bar plot of the Bayesian clustering analysis for > 1300 unique genotypes analysed at 9 microsatellite loci using STRUCTURE 2.3.4 [3]. Population ranges are abbreviated as follows: QAMA = Qamanirjuaq, CAPE = Cape Churchill, PEN = Pen Island, CHRM = Cape Henrietta Maria, FORT = Fort Severn, PEAW = Peawanuck, HARD = Harding Lake, NORW = Norway House, WAWI = Wapisu-Wimapedi, WABO = Wabowden, WHEA = Wheadon, KISS = Kississing, NARE = Naosap-Reed, BOG = The Bog, INTE = North Interlake, CHAR = Charron Lake, BERE = Berens, ATIK = Atiko, OWL = Owl-Flintstone, ATTA = Attawapiskat, BTL = Big Trout Lake, COCH = Cochrane, HEAR = Hearst, IGNA = Ignace, KAPU = Kapukasing, KEEW = Keewaywin, KENO = Kenogami, MART = Marten Falls, MOOS = Moosonee, NIPI = Nipigon, REDL = Red Lake, SIOU = Sioux Lookout, VDM – Victor Diamond Mine, WABA = Wabakimi, WEAG = Weagamow, WEBE = Webequie, WOOD = Woodland Caribou Provincial Park.

**
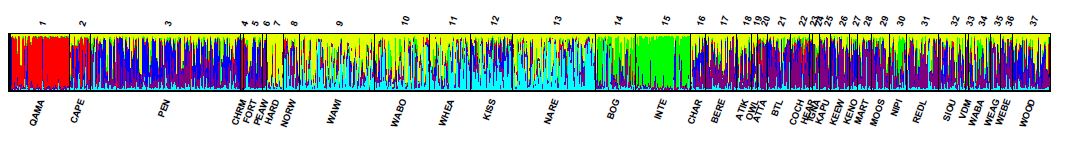
**

**Table S8.5.** Summary of genetic diversity estimates per microsatellite. Allelic size range in base pairs (bp), number of alleles (N_A_), expected (H_E_) and observed heterozygosity (H_O_), F_IS_ estimates, and standard errors (SE) for each of the estimates is given. Original references for loci are given.

| **Microsatellite Locus** | **Allelic Range** | **NA** | **H_O_** | **SE** | **H_E_** | **SE** | **F_IS_** | **SE** | **Source** |
| --- | --- | --- | --- | --- | --- | --- | --- | --- | --- |
| **BM848** | 356-390 | 17 | 0.61 | 0.05 | 0.67 | 0.05 | 0.09 | 0.03 | Bishop et al. 1994 [22] |
| **BM888** | 162-300 | 44 | 0.83 | 0.04 | 0.84 | 0.02 | 0.01 | 0.02 | Bishop et al. 1994 [22] |
| **Map2C** | 89-121 | 15 | 0.79 | 0.05 | 0.8 | 0.03 | 0.02 | 0.02 | Bishop et al. 1994 [22]  Moore et al. 1992 [24] |
| **RT5** | 88-120 | 16 | 0.8 | 0.02 | 0.82 | 0.02 | 0.03 | 0.02 | Wilson et al. 1997^*^ [20]  McLoughlin et al. 2004^**^ [21] |
| **RT6** | 88-114 | 14 | 0.72 | 0.03 | 0.74 | 0.03 | 0.02 | 0.01 | Wilson et al. 1997 [20] |
| **RT7** | 210-238 | 14 | 0.7 | 0.02 | 0.73 | 0.02 | 0.04 | 0.01 | Wilson et al. 1997 [20] |
| **RT9** | 100-130 | 16 | 0.72 | 0.03 | 0.72 | 0.03 | 0.00 | 0.01 | Wilson et al. 1997 [20] |
| **RT24** | 205-289 | 26 | 0.74 | 0.06 | 0.73 | 0.04 | - 0.01 | 0.03 | Wilson et al. 1997 [20] |
| **RT30** | 183-217 | 14 | 0.55 | 0.06 | 0.72 | 0.03 | 0.25 | 0.05 | Wilson et al. 1997 [20] |

^*^Forward primer

^**^Reverse primer.

**S8.6.** Principal Component Analysis (PCA) pre-evaluation plot generated by DIYABC version 2.0.4 [4,5] showing a representative set (10,000 simulated data set per scenario) of simulated data sets from the reference table (small dots) and the observed data (large yellow dot). As displayed, the plot shows that the observed data falls into a cloud of simulated data sets, indicating that it is possible to produce a subset of summary statistics close to the observed summary statistics with the proposed scenarios in combination with the chosen prior distributions.

S8.6.1. Pre-evaluation of microsatellite data set.


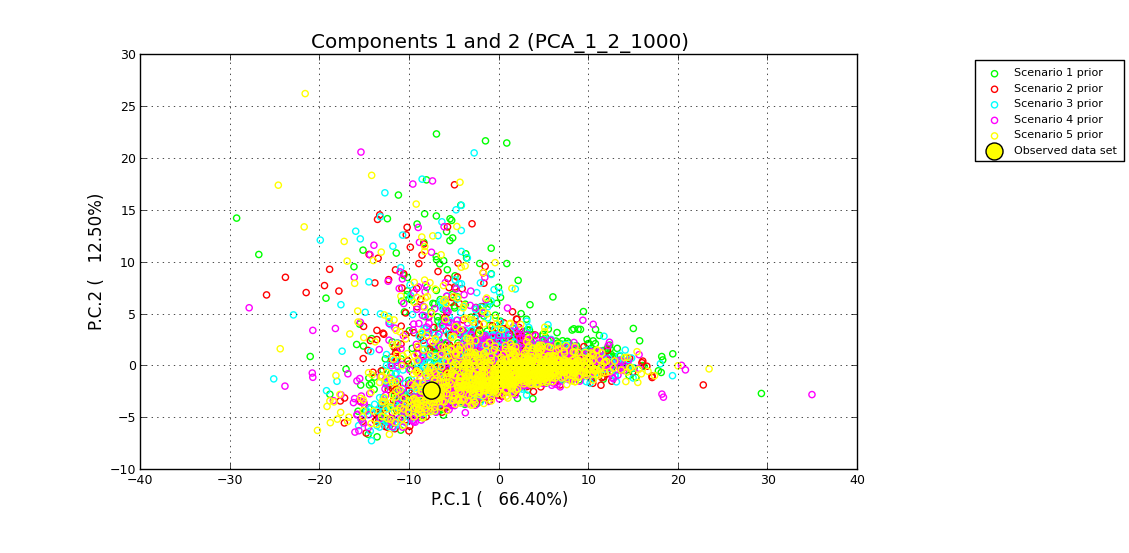


S8.6.2. Pre-evaluation of mitochondrial DNA data set.


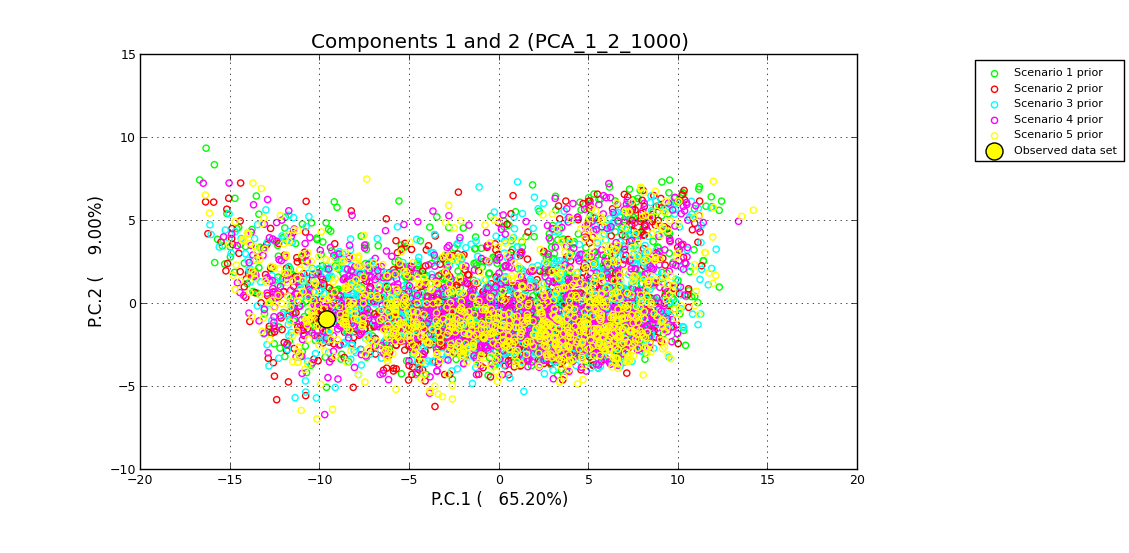


S8.6.3. Pre-evaluation of combined (i.e., microsatellites plus mitochondrial DNA) data set.


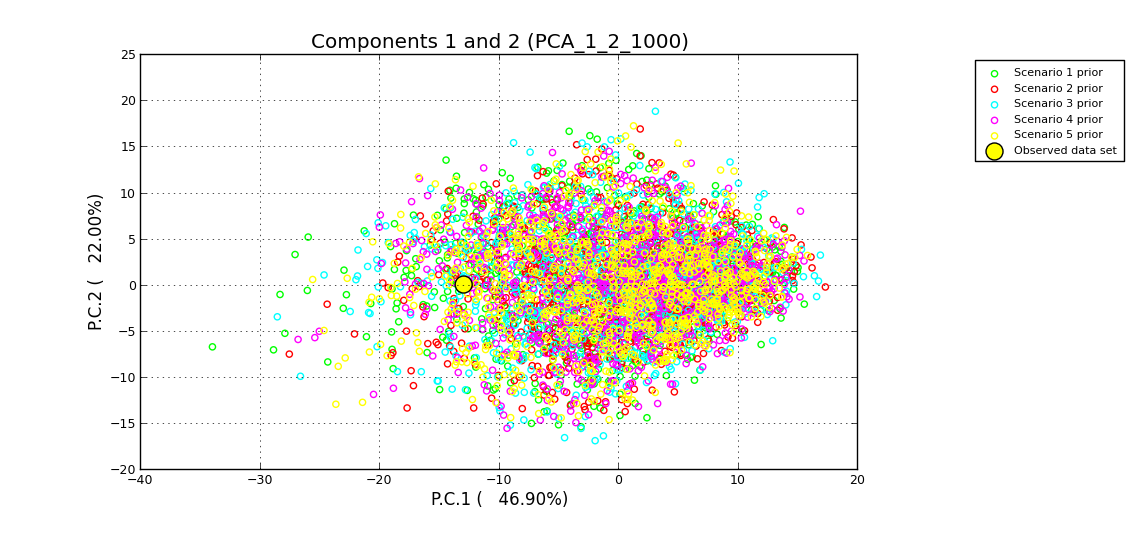


**S8.7.** Comparison of scenarios ranking the posterior probabilities for each tested scenario.

**S8.7a**. Logistic regression for microsatellite data set. Statistical support for each scenario is represented by coloured lines. Green = scenario 1, red = scenario 2, turquoise = scenario 3, light purple = scenario 4, yellow = scenario 5. Note that all scenarios except for scenario 3 are found at the bottom of the figure; thus, they are overlapping.


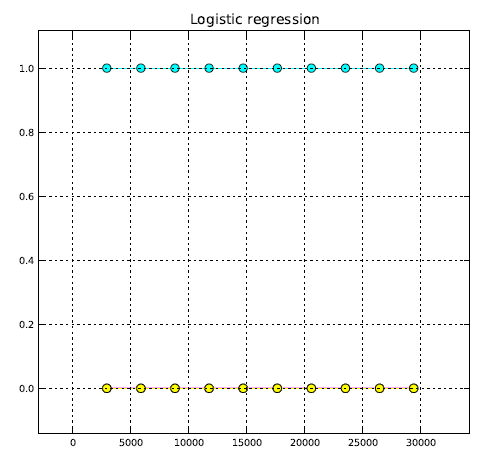


**S8.7b**. Logistic regression for mitochondrial DNA data set. Statistical support for each scenario is represented by coloured lines. Green = scenario 1, red = scenario 2, turquoise = scenario 3, light purple = scenario 4, yellow = scenario 5. Note that all scenarios except for scenario 3 are found at the bottom of the figure; thus, they are partially overlapping.


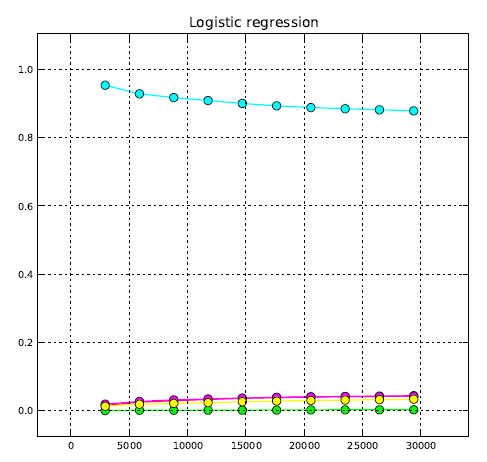


**S8.7c**. Logistic regression for combined data set. Statistical support for each scenario is represented by coloured lines. Green = scenario 1, red = scenario 2, turquoise = scenario 3, light purple = scenario 4, yellow = scenario 5. Note that all scenarios except for scenario 3 are found at the bottom of the figure; thus, they are partially overlapping.


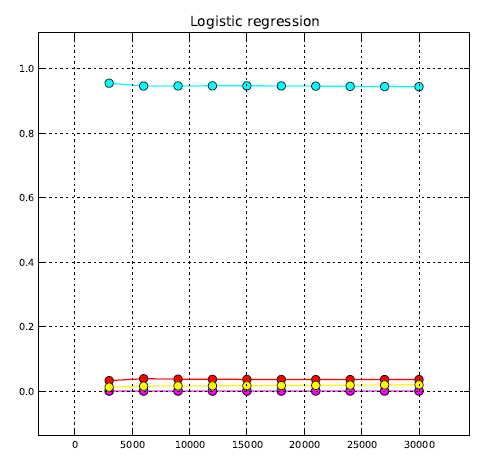


**S8.8. Time estimates – Approximate Bayesian computation (ABC)**

Time estimates for three ABC runs:

1. Microsatellite data set

| Time point | mean | median | 95% credible interval |
| --- | --- | --- | --- |
| t_1_ (gen) | 627 | 661 | 129 - 959 |
| t_1_ (years) | 4389 | 4627 | 903 - 6713 |
| t_2_ (gen) | 865 | 712 | 187 - 2410 |
| t_2_ (years) | 6055 | 4984 | 1309 - 16870 |
| t_3_ (gen) | 2100 | 2020 | 601 - 3810 |
| t_3_ (years) | 14700 | 14140 | 4207 - 26670 |
| t_4_ (gen) | 11400 | 11200 | 3800 - 19400 |
| t_4_ (years) | 79800 | 78400 | 26600 - 135800 |

1. mtDNA data set.

| Time point | mean | median | 95% credible interval |
| --- | --- | --- | --- |
| t_1_ (gen) | 139 | 72.1 | 13.6 - 677 |
| t_1_ (years) | 973 | 504.7 | 95.2 - 4739 |
| t_2_ (gen) | 2000 | 1960 | 457 - 3660 |
| t_2_ (years) | 14000 | 13720 | 3199 - 25620 |
| t_3_ (gen) | 2550 | 2790 | 317 - 3960 |
| t_3_ (years) | 17850 | 19530 | 2219 - 27720 |
| t_4_ (gen) | 10000 | 9700 | 1450 - 19300 |
| t_4_ (years) | 70000 | 67900 | 10150 - 135100 |

1. Combined data set including microsatellite and mtDNA

| Time point | mean | median | 95% credible interval |
| --- | --- | --- | --- |
| t_1_ (gen) | 426 | 413 | 84.4 - 869 |
| t_1_ (years) | 2982 | 2891 | 591 - 6083 |
| t_2_ (gen) | 972 | 835 | 240 - 2430 |
| t_2_ (years) | 6804 | 5845 | 1680 - 17010 |
| t_3_ (gen) | 1700 | 1530 | 408 - 3710 |
| t_3_ (years) | 11900 | 10710 | 2856 - 25970 |
| t_4_ (gen) | 13900 | 14300 | 6370 - 19400 |
| t_4_ (years) | 97300 | 100100 | 44590 - 135800 |

**S9. Preliminary results for Cape Churchill herd**

The Cape Churchill herd, as a putative eastern migratory ecotype, is situated between the Pen Island herd and the barren-ground distribution area (Figure S1.1). Of 27 samples, 5 (18.5%) caribou carried B haplotypes while 22 (81.5%) caribou carried A haplotypes. Thus, based on mtDNA, the Cape Churchill herd belongs to woodland caribou. Interestingly, of the 22 individuals carrying an A haplotype, 5 (22.7%) showed assignments of > 80% to barren-ground in the STRUCTURE runs based on microsatellite data. Of the 5 individuals carrying a B haplotype, 4 (80%) showed clear admixture in the STRUCTURE analysis while one was assigned to barren-ground. For Pen Island, 27 individuals carried a B haplotype (13.7 %), while 159 individuals (80.7 %) carried an A haplotype. Of those 27 individuals carrying a B haplotype, 25 (95.6 %) showed assignments of > 80% to woodland caribou. Only 2 (4.6 %) individuals carrying an A haplotype showed assignments of > 80% to barren-ground. This contrasts with the Cape Churchill herd, which is also hypothesized to be part of the eastern migratory ecotype, because the subspecies intermixing in Cape Churchill is more likely an ongoing contemporary introgressive hybridization because it can be characterized by two criteria that are commonly used to infer hybridization: 1) occurrence in an area of contact between two genetically distinct populations where hybridization is common [59] and 2) there is a possible exchange of genes between evolutionary lineages that results in viable and fertile offspring [60]. Our results from both the Pen Island and Cape Churchill herds support viable inter-breeding caribou, although Cape Churchill showed much higher contemporary gene flow in the microsatellite data set than Pen Island.

**S10. A note on hybrid swarms**

It was suggested that a postglacial ‘hybrid swarm’ of woodland and barren-ground caribou exists in the Rocky Mountains ([11] British Columbia and Alberta, Canada) and that increased migratory behaviour in some groups of mountain woodland caribou may be due to interbreeding between the two subspecies since barren-ground caribou display more migration behaviour than woodland caribou. However, the term ‘hybrid swarm’ is widely defined as a population of individuals that are all hybrids by varying numbers of generations of backcrossing with parental types and mating among hybrids [11, 44]. Unfortunately, McDevitt et al. [11] failed to provide any assessment of genetic admixture in the nuclear microsatellite data between barren-ground/Grant’s caribou and woodland caribou – most likely because no ‘pure’ B lineage group was included in the study as a reference. The presence of lineage mixing at the mtDNA level alone is insufficient to infer the level of introgression at the nuclear level and the four groups of caribou that were found with the nuclear microsatellite data set may have formed in the presence or absence of introgression. Therefore, the mechanism for interbreeding of different lineages remains unclear in western Canada.

**S11. A note on the metapopulation model**

In contrast to western Canada, Boulet et al. [57], identified that migratory and sedentary caribou herds are connected via asymmetrical gene flow in Quebec and Labrador, that is, directional gene flow from migratory to sedentary herds. This is consistent with our results that demonstrated directional gene flow from barren-ground to boreal and eastern migratory caribou. However, in contrast to the situation in Quebec and Labrador, ecotype boundaries are more defined in Manitoba. Therefore, Boulet et al. [57] proposed a metapopulation model in eastern Canada (Quebec and Labrador) for the George River and Leaf River herds (both migratory ecotype) that demonstrated high genetic exchange via calving site switching and overlapping rutting ranges. Similarly, Courtois et al. [61] suggested that the forest-dwelling caribou across the continuous range of the boreal forest are best described as a metapopulation. A metapopulation can be defined as a group of separated intra-specific local populations that interact to some extent [62] and that undergo stochastic events eventually leading to the extinction of some of local populations. Although a metapopulation model may be appropriate to characterize gene flow among herds or groups at demographic levels within higher-level groups (i.e., ecotypes, subspecies) like Boulet et al. [57] proposed, the model is unsatisfactory to describe the complex evolutionary interplay of ancient lineages in caribou across Canada and the tenet of metapopulation theory around local extinction/recolonization has not been explicitly tested across caribou ranges.

**S12. References for Electronic Supplementary Material**

1. Earl DA, vonHoldt BM. 2012 STRUCTURE HARVESTER: a website and program for visualizing STRUCTURE output and implementing the Evanno method. *Conserv. Genet. Resour.* **4**, 359 - 361. (doi: 10.1007/s12686-011-9548-7)

2. Evanno G, Regnaut R, Goudet J. 2005 Detecting the number of clusters of individuals using the software STRUCTURE: a simulation study. *Mol. Ecol.* **14**, 2611 - 2620. (doi: 10.1111/j.1365-294X.2005.02553.x)

3. Pritchard JK, Stephens M, Donnelly P. 2000 Inference of population structure using multilocus genotype data. *Genetics* **155**, 945 - 959.

4. Cornuet J-M, Pudlo P, Yeyssier J, Dehne-Garcia A, Gautier M, Lablois R, Marin J-M, Estoup A. 2014 DIYABC v2.0: a software to make approximate Bayesian computation inferences about population history using single nucleotide polymorphism, DNA sequence and microsatellite data. *Bioinformatics* **30**, 1187 - 1189. (doi: 10.1093/bioinformatics/btt763)

5. Estoup A, Lombaert E, Marin J-M, Guillemaud T, Pudlo P, Robert CP, Cornuet JM. 2012 Estimation of demo-genetic model probabilities with Approximate Bayesian Computation using linear discriminant analysis on summary statistics. *Mol. Ecol. Resour.* **12**, 846 - 855. (doi: 10.1111/j.1755-0998.2012.03153.x)

6. Hummel M, Ray JC. 2008 *Caribou and the North: A Shared Future*. Dundurn Press, Toronto.

7. Bergerud AT. 1985 Antipredator strategies of caribou: Dispersion along shorelines. *Canadian J. Zool.* **63**, 1324 - 1329.

8. Bergerud AT, Ferguson R, Butler H. 1990 Spring migration and dispersion of woodland caribou at calving. *Animal Behav.* **39**, 360 - 368.

9. Bergerud AT, Luttich SN, Camps L. 2008 The Return of Caribou to Ungava. McGill-Queen’s University Press, Montreal and Kingston.

10. Craighead JJ, Atwell G, O’Gara BW. 1972 Elk migration in and near Yellowstone National Park. Wildlife Monographs **29**, 1 - 48.

11. McDevitt AD, Mariani S, Hebblewhite M, Decesare NJ, Morgantini L, Seip D, Weckworth BV, Musiani M. 2009 Survival in the Rockies of an endangered hybrid swarm from diverged caribou (*Rangifer tarandus)* lineages. *Mol. Ecol.* **18**, 665 - 679. (doi: 10.1111/j.1365-294X.2008.04050.x)

12. COSEWIC. 2011 Designatable Units for Caribou (*Rangifer tarandus*) in Canada. Committee on the Status of Endangered Wildlife in Canada, Ottawa. 88 pp.

13. Banfield AWF. 1961 A revision of the reindeer and caribou, genus Rangifer. National Museum of Canada, Bulletin No. 177. Queen’s Printer, Ottawa. 137 pp.

14. Bergerud AT. 1996. Evolving perspectives on caribou population dynamics, have we got it right yet? *Rangifer*, Special Issue **9**, 95 - 116.

15. Couturier S, Otto RD, Cote SD, Luthier G, Mahoney SP. 2010 Body size variations in caribou ecotypes and relationships with demography. *J. Wildlife Manage.* **74**, 395 - 404. (doi: <http://dx.doi.org/10.2193/2008-384>)

16. Gunn A, Russell D, Eamer J. 2011 Northern caribou population trends in Canada. Canadian Biodiversity: Ecosystem Status and Trends 2010, Technical Thematic Report No. 10. Canadian Councils of Resource Ministers. Ottawa, ON. iv + 71 pp. <http://www.biodivcanada.ca/default.asp?lang=En&n=137E1147-1>

17. Nagy JA, Johnson DL, Larter NC, Campbell MW, Derocher AE, Kelly A, Dumond M, Allaire D, Croft B. 2011. Subpopulation structure of caribou (*Rangifer tarandus* L.) in Arctic and sub-Arctic Canada. *Ecol. Appl.* **21**, 2334 - 2348.

18. Ball MC, Pither R, Manseau M, Clark J, Petersen SD, Kingston S, Morrill N, Wilson P. 2007 Characterization of target nuclear DNA from faeces reduces technical issues associated with the assumptions of low-quality and quantity template. *Conserv. Genet.* **8**, 577 - 586. (doi: 10.1007/s10592-006-9193-y)

19. Ball MC, Finnegan L, Manseau M, Wilson P. 2010 Integrating multiple analytical approaches to spatially delineate and characterize genetic population structure: an application to boreal caribou (*Rangifer tarandus caribou*) in central Canada. *Conserv. Genet.* **11**, 2131 - 2143. (doi: 10.1007/s10592-010-0099-3)

20. Wilson GA, Strobeck C, Wu L, Coffin JW. 1997 Characterization of microsatellite loci in caribou *Rangifer tarandus*, and their use in other artiodactyls. *Mol. Ecol.* **6**, 697 - 699. (doi: 10.1046/j.1365-294X.1997.00237.x)

21. McLoughlin PD, Paetkau D, Duda M, Boutin S. 2004 Genetic diversity and relatedness of boreal caribou populations in western Canada. *Biol. Cons*. **118**, 593 - 598. (doi: 10.1016/j.biocon.2003.10.008)

22. Bishop MD *et al*. 1994 A genetic-linkage map for cattle. *Genetics* **136**, 619 - 639.

23 Stone RT, Pulido JC, Duyk GM Kappes SM, Keele JW, Beattie CW. 1995 A small-insert Bovine genomic library highly enriched for microsatellite repeat sequences. *Mamm. Genome* **6**, 714 - 724.

24. Moore SS, Barendske W, Berger KT, Armitage SM, Hetzel DJS. 1992 Bovine and ovine DNA microsatellites from the EMBL and Genbank databases. *Anim. Genet*. **23**, 410 - 414.

25. Shaw CN, Wilson PJ, White BN. 2003 A reliable molecular method of gender determination for mammals. *J. Mammal.* **84**, 123 - 128. (doi: http://dx.doi.org/10.1644/1545-1542(2003)084<0123:ARMMOG>2.0.CO;2)

26. Galpern P, Manseau M, Hettinga P, Smith K, Wilson P. 2012 Allelematch: an R package for identifying unique multilocus genotypes where genotyping error and missing data may be present. *Mol. Ecol. Resour.* **12**, 771 - 778. (doi: 10.1111/j.1755-0998.2012.03137.x)

27. Serrouya R, Paetkau D, McLellan BN, Boutin S, Campbell M, Jenkins DA. 2012 Population size and major valleys explain microsatellite variation better than taxonomic units for caribou in western Canada. *Mol. Ecol.* **21**, 2588 - 2601. (doi: 10.1111/j.1365-294X.2012.05570.x)

28. Klütsch CFC, Manseau M, Wilson PJ. 2012 Phylogeographical analysis of mtDNA data indicates postglacial expansion from multiple glacial refugia in woodland caribou (*Rangifer tarandus caribou*). *PLoS ONE* **7**, e52661. (doi: 10.1371/journal.pone.0052661)

29. Flagstad Ø, Røed KH. 2003 Refugial origins of reindeer (*Rangifer tarandus*) inferred from mitochondrial DNA sequences. *Evolution* **57**, 658 - 670. (doi: 10.1111/j.0014-3820.2003.tb01557.x)

30. Hall TA. 1999 BioEdit: a user-friendly biological sequence alignment editor and analysis program for Windows 95/98/NT. *Nucleic Acids S.* **41**, 95 - 98.

31. Librado P, Rozas J. 2009 DnaSP v5: A software for comprehensive analysis of DNA polymorphism data. *Bioinformatics* **25**, 1451 - 1452. (doi: 10.1093/bioinformatics/btp187)

32. van Oosterhout C, Hutchinson WF, Wills DP, Shipley P. 2004 Micro-Checker: software for identifying and correcting genotyping errors in microsatellite data. *Mol. Ecol. Resour.* **4**, 535 -538. (doi: 10.1111/j.1471-8286.2004.00684.x)

33. Rousset F. 2008 GenePop’007: a complete re-implementation of the GenePop software for Windows and Linux. *Mol. Ecol. Resour.* **8**, 103 - 106. (doi: 10.1111/j.1471-8286.2007.01931.x)

34. Weir BS, Cockerham CC. 1984 Estimating F-statistics for the analysis of population structure. *Evolution* **38**, 1358 - 1370.

35. Kalinowski ST. 2004 Counting alleles with rarefaction: private alleles and hierarchical sampling designs. *Conserv. Genet.* **5**, 539 - 554. (doi: 10.1023/B:COGE.0000041021.91777.1a)

36. Kalinowski ST. 2005 HP-Rare: a computer program for performing rarefaction on measures of allelic diversity. *Mol. Ecol. Notes* **5**, 187 - 189. (doi: 10.1111/j.1471-8286.2004.00845.x)

37. Peakall R, Smouse PE. 2012 GenAlEx 6.5: genetic analysis in Excel. Population genetic software for teaching and research – an update. *Bioinformatics* **28**, 2537 - 2539. (doi: 10.1093/bioinformatics/bts460)

38. Excoffier L, Lischer HEL. 2010 Arlequin suite ver 3.5: A new series of programs to perform population genetics analyses under Linux and Windows. *Mol. Ecol. Resour.* **10**, 564 - 567. (doi: 10.1111/j.1755-0998.2010.02847.x)

39. Falush D, Stephens M, Pritchard JK. 2003 Inference of population structure: extensions to linked loci and correlated allele frequencies. *Genetics* **164**, 1567 - 1587.

40. Jakobsson M, Rosenberg NA. 2007 *CLUMPP*: a cluster matching and permutation program for dealing with label switching and multimodality in analysis of population structure. *Bioinformatics* **23**, 1801 - 1806. (doi: 10.1093/bioinformatics/btm233)

41. Rosenberg N. 2004 DISTRUCT: a program for the graphical display of population structure. *Mol. Ecol. Notes* **4**, 137 - 138. (doi: 10.1046/j.1471-8286.2003.00566.x)

42. Vähä JP, Primmer CR. 2006 Efficiency of model-based Bayesian methods for detecting hybrid individuals under different hybridization scenarios and with different numbers of loci. *Mol. Ecol.* **15**, 63 - 72. (doi: 10.1111/j.1365-294X.2005.02773.x)

43. Bohling JH, Waits LP. 2011 Assessing the prevalence of hybridization between sympatric *Canis* species surrounding the red wolf (*Canis rufus*) recovery area in North Carolina. *Mol. Ecol*. **20**, 2142 - 2156. (doi: 10.1111/j.1365-294X.2011.05084.x)

44. Latch EK, Kierepka EM, Heffelfinger JR, Rhodes JR OE. 2011 Hybrid swarm between divergent lineages of mule deer (*Odocoileus hemionus*). *Mol. Ecol.* **20**, 5265 - 5279. (doi: 10.1111/j.1365-294X.2011.05349.x)

45. Csilléry K. *et al.* 2010 Approximate Bayesian Computation (ABC) in practice. *Trends Ecol. Evol*. **25**, 410 - 418. (doi: 10.1016/j.tree.2010.04.001)

46. Beaumont MA. 2010 Approximate Bayesian Computation in Evolution and Ecology. *Annu. Rev. Ecol. Evol. Syst*. **41**, 379 - 406. (doi: 10.1146/annurev-ecolsys-102209-144621)

47. Inoue K, Monroe EM, Elderkin CL, Berg DJ. 2014 Phylogeographic and population genetic analyses reveal Pleistocene isolation followed by high gene flow in a wide ranging, but endangered, freshwater mussel. *Heredity* **112**, 282 - 290. (doi: 10.1038/hdy.2013.104)

48. Stockwell C, Heilveil JS, Purcell K. 2013 Estimating divergence time for two evolutionary significant units of a protected fish species. *Conserv. Genet*. **14**, 215 – 222. (doi: 10.1007/s10592-013-0447-1)

49. Meraner A, Cornetti L, Gandolfi A. 2014 Defining conservation units in a stocking-induced genetic melting plot: unraveling native and multiple exotic genetic imprints of recent and historical secondary contact in Adriatic grayling. *Ecol. Evol*. **4**, 1313 – 1327. (doi: doi: 10.1002/ece3.931)

50. Matocq MD, Kelly PA, Phillips SE, Maldonado JE. 2012 Reconstructing the evolutionary history of an endangered subspecies across the changing landscape of the Great Central Valley of California. *Mol. Ecol*. **21**, 5918 – 5933. (doi: 10.1111/mec.12079)

51. Phillips CD, Hoffman JI, George JC, Suydam RS, Huebinger RM, Patton JC, Bickham JW. 2013 Molecular insights into the historic demography of bowhead whales: understanding the evolutionary basis of contemporary management practices. *Ecol. Evol.* **3**, 18 - 37. (doi: 10.1002/ece3.374)

52. Darriba D, Taboada GL, Doallo R, Posada D. 2012 jModelTest 2: more models, new heuristics and parallel computing. *Nature Methods* **9**, 772. (doi: 10.1038/nmeth.2109)

## 53. Yannic G *et al.* 2014 Genetic diversity in caribou linked to past and future climate change. *Nature Clim. Change* 4, 132 - 137. (doi: 10.1038/nclimate2074)

54. COSEWIC. 2002 COSEWIC assessment and update status report on the Woodland caribou *Rangifer tarandus caribou* in Canada. Ottawa, pp 98.

55. Fuller TK, Keith LB. 1981 Woodland caribou population dynamics in northeastern Alberta. *J. Wildlife Manage.* **45**, 197 - 213. (doi: 10.2307/3807887)

56. Adams LG, Dale W. 1998 Reproductive performance of female Alaskan caribou. *J. Wildlife Manage.* **62**, 1184 - 1195. (doi: 10.2307/3801982)

57. Boulet M, Couturier S, Côté SD, Otto RD, Bernatchez L. 2007 Integrative use of spatial, genetic, and demographic analyses for investigating genetic connectivity between migratory, montane, and sedentary caribou herds. *Mol. Ecol.* **16**, 4223 - 4240. (doi: 10.1111/j.1365-294X.2007.03476.x)

58. Cornuet JM, Santos F, Beaumont MA, Robert CP, Marin JM, Balding DJ, Guillemaud T, Estoup A. 2008 Inferring population history with DIY ABC: a user-friendly approach to approximate Bayesian computation. *Bioinformatics* **24**, 2713 - 2719. (doi:10.1093/bioinformatics/btn514)

59. Allendorf FW, Leary RF, Spruell P, Wenburg JK. 2001 The problems with hybrids: setting conservation guidelines. *Trends Ecol. Ecol.* **16**, 613 - 622. (doi: [doi:10.1016/S0169-5347(01)02290-X](http://dx.doi.org/10.1016/S0169-5347(01)02290-X))

60. Seehausen O. 2004 Hybridization and adaptive radiation. *Trends Ecol. Evol.* **19**, 198 - 207. (doi: [10.1016/j.tree.2004.01.003](http://dx.doi.org/10.1016/j.tree.2004.01.003))

61. Courtois R, Bernatchez L, Ouellet J–P, Breton L. 2003 Significance of caribou (*Rangifer tarandus*) ecotypes from a molecular viewpoint. *Conserv. Genet.* **4**, 393 - 404. (doi: 10.1023/A:1024033500799)

62. Levins R. 1969 Some demographic and genetic consequences of environmental heterogeneity for biological control. *Bull. Entomol. Soc. America* **15**, 237 - 240.
